# Development and evaluation of a package to improve hypertension control in Nigeria [DEPIHCON]: a cluster-randomized controlled trial

**DOI:** 10.1186/s13063-022-06209-9

**Published:** 2022-05-02

**Authors:** IkeOluwapo O. Ajayi, Oyediran E. Oyewole, Okechukwu S. Ogah, Joshua O. Akinyemi, Mobolaji M. Salawu, Eniola A. Bamgboye, Taiwo Obembe, Morenikeji Olawuwo, Mahmoud Umar Sani

**Affiliations:** 1 Epidemiology and Biostatistics Research Unit, Institute of Advanced Medical Research and Training, College of Medicine, University of Ibadan, Ibadan, Nigeria; 2 Department of Epidemiology and Medical Statistics, Faculty of Public Health, College of Medicine, University of Ibadan, Ibadan, Nigeria; 3 Department of Health Promotion and Education, Faculty of Public Health, College of Medicine, University of Ibadan, Ibadan, Nigeria; 4 Department of Internal Medicine, Faculty of Clinical Sciences, College of Medicine, University of Ibadan, Ibadan, Nigeria; 5 Department of Health Policy and Management, Faculty of Public Health, College of Medicine, University of Ibadan, Ibadan, Nigeria; 6 Department Internal Medicine, Aminu Kano Teaching Hospital, Kano, Nigeria

**Keywords:** Hypertension, Hypertension risk factors, Community-oriented resource persons, Healthy lifestyle promotion, Task shifting, Blood pressure control, Randomized controlled trial, Cluster

## Abstract

**Background:**

Nigeria’s healthcare system capacity to stem the increasing trend in hypertension is limited in coverage, scope and manpower. Use of trained community-based care providers demonstrated to be an effective complement in improving access to, and supporting healthcare delivery has not been adequately examined for hypertension care in Nigeria. This study is proposed to evaluate the effectiveness of using trained community-oriented resource persons (CORPs) to improve hypertension control in Nigeria.

**Methods:**

An intervention study will be conducted in three states using a mixed method design. First is a baseline survey using a semi-structured pre-tested questionnaire to collect information on demographics, clinical data, knowledge, occurrence and risk factors of hypertension among 1704 adults ≥18 years. Focus group discussions (FGD) and key informant interviews (KII) will be conducted to explore a community’s experience of hypertension, challenges with hypertension management and support required to improve control in 10 selected communities in each state. The second is a cluster-randomized controlled trial to evaluate effect of a package on reduction of blood pressure (BP) and prevention of cardiovascular (CVD) risk factors among 200 hypertensive patients to be followed up in intervention and control arms over a 6-month period in each state. The package will include trained CORPs conducting community-based screening of BP and referral, diagnosis confirmation and initial treatment in the health facility, followed by monthly home-based follow-up care and provision of health education on hypertension control and healthy lifestyle enhanced by phone voice message reminders. In the control arm, the usual care (diagnosis, treatment and follow-up care in hospital of a patient’s choice) will continue. Third, an endline survey will be conducted in both intervention and control communities to evaluate changes in mean BP, control, knowledge and proportion of other CVD risk factors. In addition, FGD and KII will be used to assess participants’ perceived quality and acceptability of the interventions as delivered by CORPs.

**Discussion:**

This research is expected to create awareness, improve knowledge, perception, behaviours, attitude and practices that will reduce hypertension in Nigeria. Advocacy for buy-in and scale up of using CORPs in hypertension care by the government is key if found to be effective.

**Trial registration:**

PACTR Registry PACTR202107530985857. Registered on 26 July 2021.

## Administrative information

Note: the numbers in curly brackets in this protocol refer to SPIRIT checklist item numbers. The order of the items has been modified to group similar items (see http://www.equator-network.org/reporting-guidelines/spirit-2013-statement-defining-standard-protocol-items-for-clinical-trials/).

**Table:** 

Title {1}	Development and evaluation of a package to improve hypertension control in Nigeria [DEPIHCON]: study protocol for a cluster randomized controlled trial
Trial registration {2a and 2b}.	PACTR Registry- PACTR202107530985857 Registered on 26 July 2021 Url – https://pactr.samrc.ac.za
Protocol version {3}	December 01, 2019; version number is 3
Funding {4}	Tertiary Education Trust Fund (TETFund), National Research Fund, Nigeria [TETFund/DR&D/CE/NRF/STI/43/VOL1]
Author details {5a}	Prof IkeOluwapo O. Ajayi - Epidemiology and Biostatistics Research Unit, Institute of Advanced Medical Research and Training, College of Medicine, University of Ibadan Dr Joshua O. Akinyemi – Department of Epidemiology and Medical Statistics, Faculty of Public Health, College of Medicine, University of Ibadan Dr Mobolaji M. Salawu - Department of Epidemiology and Medical Statistics, Faculty of Public Health, College of Medicine, University of Ibadan Dr Eniola A. Bamgboye - Department of Epidemiology and Medical Statistics, Faculty of Public Health, College of Medicine, University of Ibadan Dr Oyediran E. Oyewole - Department of Health Promotion and Education, Faculty of Public Health, College of Medicine, University of Ibadan Dr Okechukwu S. Ogah – Department of Internal Medicine, Faculty of Clinical Sciences, College of Medicine, University of Ibadan Prof Mahmoud U Sani – Department of Internal Medicine, Aminu Kano Teaching Hospital, Kano, Nigeria Dr Taiwo Obembe - Department of Health Policy and Management, Faculty of Public Health, College of Medicine, University of Ibadan
Name and contact information for the trial sponsor {5b}	Prof. Suleiman E. Bogoro The Director Research and Development /Centres of Excellence Tertiary Education Trust Fund No 6, Zambezi Crescent, Maitama, Abuja Telephone No.: + 2348008383863 E-mail: rdcedept@tetfund.gov.ng
Role of sponsor {5c}	The funders reviewed the proposal at the selection stage and provide useful suggestions to include to meet the mandate of the funding agency. The funders had no role in the study design, collection and management, analysis and interpretation of data. However, the funders will carry out monitoring visits periodically.

## Introduction

### Background and rationale {6a}

Hypertension is now recognized as a major contributor to the global burden of diseases and mortality [1, 2]. The prevalence of hypertension is rising globally owing to demographic transition and increase in exposure to lifestyle risk factors including unhealthy diets such as high sodium, low potassium intake and lack of physical activity [3].

An estimated 1.13 billion people worldwide have hypertension with, two-thirds living in low- and middle-income countries [4]. .Estimates suggest that in 2010, 31.1% of adults worldwide had hypertension. The prevalence of hypertension among adults was higher in LMICs than in high-income countries (31.5% versus 28.5%) [3]. In 2015, an estimated 8.5 million deaths were attributable to systolic blood pressure > 115 mmHg, 88% of which were in low-income and middle-income countries [5]. While average age-standardized BP is decreasing in most high-income countries, it is increasing in most low-income and middle-income countries with 32%–50% of adults estimated to be hypertensive in sub-Saharan Africa [3].

Hypertension is a leading risk of death in sub-Saharan Africa causing more than half a million deaths and more than 10 million life years lost. The high prevalence of hypertension and the increasing trend of early onset is a major concern in the development of the region as the young and active workforce population are being incapacitated and worst still depleted. Reports from WHO in 2015 shows the region currently has the highest age- standardized rates of hypertension worldwide with about 46% of individuals aged 25-years and above being hypertensive [6].

In Nigeria, like in most African countries, hypertension is the foundation of cardiovascular disease [7]. In a recent meta-analysis of blood pressure surveys in Nigeria, the documented overall prevalence of hypertension was 28.9% (95%CI, 21.1–32.8%), with a prevalence of 29.5% (95%CI, 24.8–34.3%) among men and 25.0% (95%CI, 20.2–29.7%) among women [8]. There were about 20 million cases of hypertension in the country in the year 2010. This is projected to rise to 39.1 million cases by the year 2030 [8]. Currently, an epidemic of premature cardiovascular mortality and early appearance of hypertension, a risk factor, is being experienced in Nigeria [9, 10]. Studies conducted by our team in two urban communities in Ibadan, which constitutes one of the study sites (Oyo State) for this proposed study, found prevalence of hypertension to range from 20.4 [9] to 33.1% [11] in adults.

Hypertension and its complications are responsible for about 25% of emergency medical admissions in urban hospitals in Nigeria [12, 13]. However, the level of awareness of its detrimental effect is abysmally low [8] and contributed to the poor control of the disease. The Prospective Urban Rural Epidemiology study showed that despite high levels of hypertension worldwide, only 34% of Africans are aware of their hypertension status, only 31.3% receive any treatment and only 6.5% have their BP under control [14].

In Nigeria, treatment and control of hypertension is poor. A study conducted among hypertensives attending health facilities in Federal Capital Territory reported about 70% of patients were non-adherent to treatment [15] while another study in Northern Nigeria reported a similar proportion of hypertensives did not achieve blood pressure control [16]. Factors adduced for this poor outcome of treatment include those related to patients such as non-affordability of treatment cost, inadequate awareness and poor knowledge of hypertension, poor health-seeking behaviour, low compliance with scheduled follow-up visits and suboptimal pharmacotherapy and lack of trust in the healthcare system among others. Health system-related factors include lack of infrastructure/equipment/medicines, poor attitude, remuneration of health workers, lack of skilled/trained manpower, non-inclusion of hypertension treatment in primary healthcare function and inadequate facilities in the community [17]. The other factors include poverty, unhealthy dietary intake and sedentary lifestyle [17].

Currently there is a paradigm shift in the control of hypertension in line with community-based approach to the control of diseases, termed Community-clinical linkages, which is becoming a complementary strategy to existing health facility-based interventions in countries with limited health manpower and in remote communities. Community-clinical linkages are connections between community and clinical sectors to improve population health, foster community-clinic relationship and improve patient’s access to preventive and chronic care services [18]. This strategy has been used successfully in control of infectious diseases [references - proposal [19–21]], and the use in hypertension control is gaining ground in sub-Saharan Africa with some demonstration of promising benefits in reducing hypertension and changes in behaviour related to healthy lifestyle when CHWs implement community-based interventions [22–25]. A few interventions may be ongoing [26], and this proposed study stands to learn voice on /contribute to knowledge on the effectiveness of this strategy and share experience as conducted in Nigeria.

In Nigeria, CORPs are part of community-based care providers who are called differently as community health works, village health workers, village/community volunteers and community-based drug distributors among others depending on the setting. The use of this group of people in task sharing in Nigeria varies depending on location and need. In Nigeria, some lay persons who met stipulated criteria have been trained for this function and used in communities for different assignments such as for immunization campaign, mobilizers, health education, drug distribution, integrated community-based case management iCCM of febrile illness [27] and home management of malaria [26]. The use of community-based volunteers has also been carried out for interventions geared towards reduction of maternal deaths facilitating early presentation in pregnancy and adherence to clinic visits [28]. Furthermore, the strategy has been used under community-directed interventions (CDI) for ivermectin distribution for onchocerciasis and later for malaria and tuberculosis treatment [29].

## Conceptual framework

Precede-Proceed framework was considered suitable for the implementation of this project. This is because it focuses on three cardinal issues, which include the Predisposing, Enabling and Reinforcing factors. PRECEDE stands for Predisposing, Reinforcing, and Enabling Constructs in Educational Diagnosis and Evaluation. It involves social, epidemiological, ecological factors assessments; assessing the following community factors: PROCEED stands for Policy, Regulatory, and Organizational Constructs in Educational and Environmental Development. The issues concerning the individuals, community and the health system are well accentuated in the framework. The variables in the specific objectives of the projects are well captured in the framework. The PRECEDE-PROCEED model is a comprehensive structure for assessing health needs for designing, implementing and evaluating health promotion and other public health programmes to meet those needs [30]. PRECEDE provides the structure for planning a targeted and focused public health programme while PROCEED provides the structure for implementing and evaluating the public health programme.

### Objectives {7}

The study aims to evaluate the effectiveness of an intervention package on knowledge and control of high blood pressure, and reduction in hypertension risk factors among adults. The acceptability of CORPs and their effectiveness in delivering the intervention will also be assessed.

### Trial design {8}

**Fig. 1:**
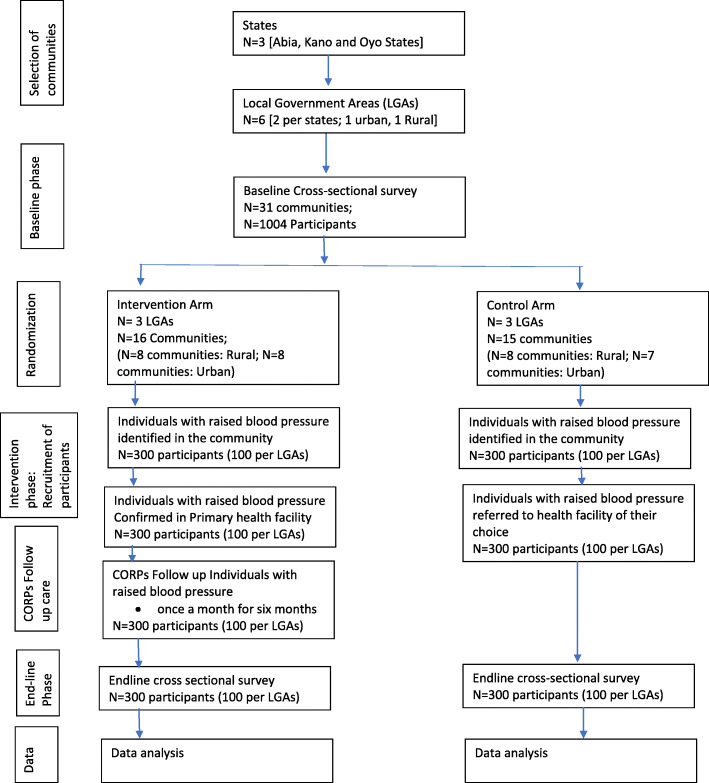
Study design and flow chart

This study will employ a cluster-randomized controlled trial and mixed method of data collection. It comprises of three phases: baseline, intervention and post-intervention: Baseline and endline surveys which will be conducted after 6 months of intervention in the control and intervention arms of the study. Information will be collected on socio-demographic variables, anthropometry measurements such as weight and height, clinical data and biochemical analysis of food sodium content, urinary salt and serum lipid profiles. Focus group discussion (FGD) for community members and key informant interviews (KII) for stakeholders at the health facilities and in the community will be conducted. A follow-up study will be implemented in the intervention arm deploying the intervention over a 6-month period after which effect on hypertension control and fidelity of implementation will be evaluated. The study design and flow chart are shown in Fig. 1.

## Methods: participants, interventions and outcomes

### Study setting {9}

The study will be conducted in three states selected from the six geopolitical zones in Nigeria comprising of Abia (South East), Kano (North West) and Oyo (South West) states. A state in Nigeria comprises a number of LGAs and each LGA is made up of 10 to 12 wards.

**Fig. 2:**
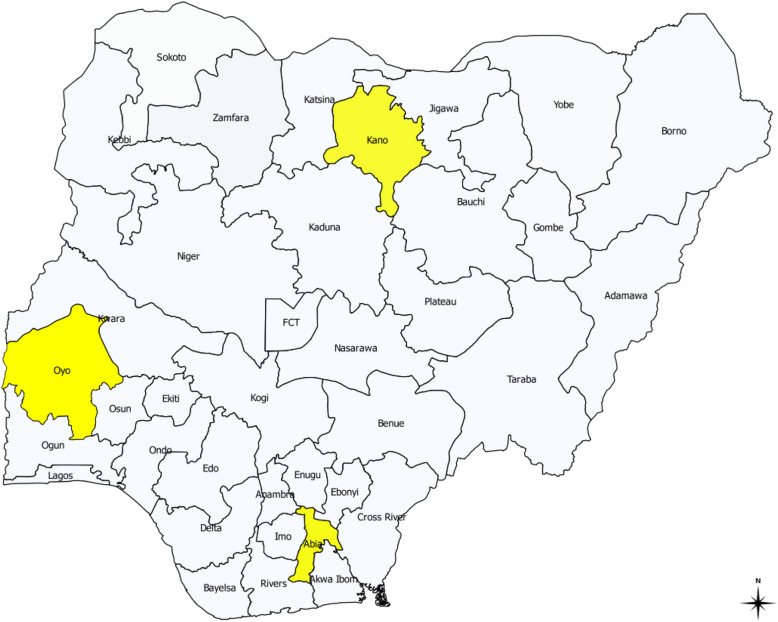
Map of Nigeria showing the states selected for study

Two Local Government Areas (LGAs) (one urban and one rural) each were selected from each state based on participation in past hypertension survey and good cooperation received from the community members in past studies. Thus, in Abia State—Umuahia North (urban) and Isuikwuato (rural) with population of 292,300 and 151,700; in Kano state—Tarauni (Urban) and Bichi (Rural) with population of 387,100 and 308,600; and in Oyo state—Ibadan North (Urban) and Ona-Ara (Rural) with population of 473,900 and 373,100 respectively will be studied [31]. Figure 2 is the map of Nigeria showing selected states visited.

In the urban sites, the predominant economic activities are white- and blue-collar jobs, trading and other commercial activities. There are small-, medium- and large-scale industries and very little farming activities, whereas, in the rural sites, the people are predominantly farmers with few small-scale industries and trading.

The Local Government in each of the states has a Secondary/Comprehensive health facility, while each ward has a primary health facility (PHC). The PHCs are manned by a team of medical officer, nurses, community health extension workers, junior community health extension workers and pharmacy assistants headed by the medical officer or senior nurse where there is no medical officer.

Existing within the wards are trained lay persons who have served as community health workers at some point or participated in previous community-based studies such as the home management of malaria using artemisinin-based combination therapy study. Some will be selected from them to serve as CORPs. Where there are no CORPs, communities shall nominate persons who meet selection criteria to represent their communities.

### Eligibility criteria {10}

The study will involve two groups of participants: the community members and the CORPs.

#### Community members

The community members will participate at the three phases of the study.

For the base- and endline surveys, participants will be consenting residents of the community aged ≥18 years. A resident will be someone who has been living in the community for 6 months or more. Those who are sick at time of survey, mentally challenged or have communication challenge and pregnant women will be excluded from the survey. In addition, at endline survey, a subgroup of participants in the intervention arm will be randomly selected to participate in focus group discussions and in-depth interviews to explore their acceptability and perception of quality of the CORPs delivered follow-up care, as well as perceived challenges to implementation and sustainability.

For recruitment into the intervention, those found at community screening to have elevated blood pressure and same confirmed in the health facility, old patients receiving treatment and not controlled or not on treatment will be eligible for recruitment. They must plan to reside in the community for at least 1 year after enrollment and willing to participate in the home-based follow-up care by CORPs. To be excluded are patients with clinically established complications and other cardiovascular diseases, patients needing or on special medical services and pregnant women. The health workers to recruit at the health facilities will be medical officers of health and nurses involved in the care of adults in the health facilities. They will be trained on diagnosis, treatment and prevention of hypertension as well as use of the national guideline for treatment of hypertension.

#### Community-oriented resource persons (CORPs)

The set of inclusion criteria to be met by any persons to be nominated by the community to participate as a CORP include the following: (i) an adult > 18 years (male or female], (ii) have completed at least primary level education with ability to read and write, (iii) may have worked as a volunteer on community project especially in the area of health and must have performed well while serving on the project, (iv) may be an unemployed healthcare worker such as trained junior community health worker (JCHEWs), or retired nurse/healthcare professional, retired civil servant with interest in providing care, (v) where married, the spouse must be supportive, (vi) must be acceptable to other members of their community, (vii) must have been resident in the community for at least one year. Two nominees will be from each community. The nominated members will participate in a 4-day training and the final selection of CORPs to participate in the study will be based on their performance at the training which will be assessed using pre- and post-training test and score at practical exercises.

### Who will take informed consent? {26a}

Informed consent will be obtained at the different level/phases of implementation. For the baseline study, informed consent will be taken from participants by the interviewing research assistants during the survey and qualitative studies. At the health facility, informed consent will be obtained from those found to be hypertensive and eligible to be recruited including consent to receive home-based follow-up care.

### Additional consent provisions for collection and use of participant data and biological specimens {26b}

This trial involves collecting biological specimens for storage. The informed consent form will have a paragraph seeking for the later use of data collected (survey data and blood samples) with assurance of confidentiality.

## Interventions

### Explanation for the choice of comparators {6b}

Comparators will be adults aged 18 years and above who are living in the control communities and receiving usual care for the management of hypertension.

### Intervention description {11a}

The highlights of the activities and assessments for intervention and control arm are shown in Table 1. The components of the intervention include the following.

**Table 1:** Highlights of the activities and assessments for intervention and control arm

	Activity	Intervention	Control
1	Baseline survey	Yes	Yes
2	Selection, screening and standard training of CORPs on blood pressure screening	Yes	Yes
3	Training on additional information on follow-up care for hypertensive patients and CORPs coordination	Yes	No
4	Trained CORPs screen and refer community members found to have high blood pressure	Yes Referral to the designated primary healthcare centre in the community	Yes Referral will be to the community member’s health facility of choice
5	Train health facility staff in designated primary healthcare facilities on the National guideline for hypertension treatment	Yes	No
6	CORPs Monthly checking and recording of blood pressure as well as providing health education/counselling support by the CORPs	Yes	No
7	SMS messaging on medication and healthy lifestyle adherence to support follow-up care	Yes	No
8	Anthropometry measurement, e.g. weight, height and body mass index (BMI) Biochemical measurement ECG	Yes	No
9	Endline survey	Yes	Yes

#### i. Training of CORPs

A 4-day training will be conducted for selected persons to serve as CORPs using a training manual developed by the team. A pre- and post-test will be conducted to assess the knowledge gained during the training and to select those with high score and practical ability who will eventually participate in the project. At the training, draft health education materials and messages shall be revised with input by the CORPs and finalized for use (participatory approach). A refresher training will be conducted 6 weeks into the intervention and on the job refresher training shall be conducted by the supervisors as found necessary.

#### ii. Community-based education on hypertension, risk factors, complications and healthy lifestyle

The CORPs will provide health education to raise awareness on hypertension, risk factors, importance of screening, adherence to medication and healthy lifestyle practices in the community and during home visits. They will also mobilize the community to come out for screening. The pre-tested health education materials will be used to facilitate the community health education and will be distributed to the participants for their reference.

#### iii. Community-based screening, referral and linkage to health facility

The CORPs will be equipped with a tool kit consisting an OMRON digital sphygmomanometer, a weighing scale, tape rule and recording forms. They will visit homes, religious houses and organizational meetings in the community to screen for hypertension. In the intervention arm, trained CORPs will screen and refer individuals found to have high blood pressure to the primary healthcare centre in the community. Referral forms will be provided.

#### iv. Initial treatment and counselling of individuals confirmed to have hypertension in a health facility

Health workers will be provided and trained on application of the national guideline for hypertension treatment being recommended by the Federal Ministry of Health for primary health facilities across the country. At the health facility, the attending physician/health worker will confirm diagnosis of hypertension among individuals referred by the CORPs and those found to be hypertensive among persons visiting the health facility for other reasons or self-referred. The physician/health worker will commence antihypertensive medications based on the National guideline for the treatment of hypertension and provide initial counselling on medication adherence and healthy lifestyle. Thereafter, the patient will be referred back to the CORP in their community for home-based follow-up care. The patients in the intervention sites will be given 1-month clinic follow-up appointment at first visit to ascertain the follow-up process is in place, then every 2 months thereafter by the attending physician/healthcare worker. However, the physicians can use their discretions depending on the clinical status of the patient. A SMS voice messaging for treatment adherence, reminders and health messaging will be sent to participants weekly [32]

#### iv. Community-based hypertension follow-up care by CORPs

The CORPs will conduct routine follow-up patients in their homes monthly checking their blood pressure, adherence to medication and providing health education/counselling support. In addition, the CORPs will ask about their attendance to health facility as they were scheduled/advised and sought reasons for non-attendance among those who may not have adhered. The CORP will organize group lifestyle education session weekly where experiences will be shared and patients will engage in group exercises among other relevant activities. Follow-up record form will be used by CORPs to detail information on patients’ compliance with drug, the health education provided and BP measurements. A CORP will be attached to 20 hypertensive patients for follow-up care. The intervention will be for 6 months.

### Criteria for discontinuing or modifying allocated interventions {11b}

Any patient in the intervention arm who does not achieve blood pressure control over 2 follow-up visits by the CORPS or who develop complication of hypertension or found to be at risk of complication based on risk assessment may be withdrawn from the study after investigators review the case. Any CORP found not to perform effectively will be disengaged.

### Strategies to improve adherence to interventions {11c}

To enhance adherence to the intervention, we have proposed the following strategies: (i) short message services on healthy lifestyle and medication adherence which will be sent to patients weekly; monthly monitoring of CORPS by supervisors, and pill counting by CORPs at home follow-up visits. There is a plan to engage drug pharmaceuticals to supply antihypertensives to patients at discounted price as much as possible.

### Relevant concomitant care permitted or prohibited during the trial {11d}

This trial involves screening, diagnosis and treatment of hypertension. The control arm will be on the standard/usual care while the intervention will have in addition to standard care follow-up care at home by trained community-oriented resource person. This is an implementation research which will be carried out in a real-life situation. There will be no concomitant care and participants will be advised to take only medications prescribed in health facilities or report any other medication that may have been taken during the period in the trial.

### Provisions for post-trial care {30}

No harm is envisaged from this trial.

### Outcomes {12}

The Primary outcomes are (i) change in baseline proportion of patients with controlled blood pressure, (ii) the mean change in blood pressure at 6 months between patients in the intervention and control communities. The secondary outcome measures are (i) change from baseline in proportion of patients adhering to medication, (ii) change in proportion of patients who are overweight/obese, smoke, take alcohol, practice unhealthy dietary habit and are physically inactive. In addition are (iii) mean improvement in quality-of-life score, (iv) percentage increase in satisfaction with care score, (v) change in awareness, knowledge, perception and practice, (vi) proportion of CORPs assessed to have good competency score, (vii) perception about quality and acceptability of home-based follow-up care delivered by CORPs, (viii) incidence of cardiovascular events among patients, (ix) change in baseline proportion of patients assessed to have high cardiovascular risk, (x) mean cost of treatment of hypertension, (xi) mean change in lipid level, urinary sodium and food sodium content. These variables will be measured at the end of the second, fourth and sixth month follow-up period.

### Participant timeline {13}

The project timeline showing schedule of enrolment, intervention and assessment is shown in Table 2. The project is for a 2-year period which has been divided into eight quarters and activities for each quarter indicated and the corresponding year.

**Table 2:** The project timeline

S/N	Description of activity	Duration	Year	Quarter							
				1st	2nd	3rd	4th	5th	6th	7th	8th
1	Formation of team and coordination Planning and need assessment	3 months	1	X							
2	Finalizing protocol and manuals	2 months	1		X						
3	Hiring staff; selection and training of CORPs; identification of health Facilities and training of health workers	1 month	1		X						
4	Baseline studies and data analysis and interim report	2 months	1		X						
5	Preparation for intervention, Development of IEC	1 month	1		X						
6	Recruitment of patients	3 months	1			X					
7	Conducting follow-up visits	6 months	1 & 2				X	X			
8	Laboratory samples collected		1 & 2			X			X		
9	Mid-study report	1 month	1				X				
10	Post-intervention survey	3 months	2							X	
11	Final data analysis	2 months	2							X	
12	Final report writing	1 months	2								X

### Sample size {14}

The sample size will be determined using the superiority trial formula [33], power of 90% (*Z*_β_ = 1.28) and standard normal deviate (*Z*_α/2 =_ 1.96) corresponding to a 95% level of confidence.

Current literature indicates that only 30.0% [18] of those living with hypertension are controlled (p_1_). It is anticipated that the implementation of the intervention package will raise the percentage to 60.0% (p_2_). Based on these parameters, and adjusting for 10.0% attrition, a sample size of 50 persons living with hypertension will be required for each arm. This translates to 100 per LGA and 200 per state. Overall, 600 participants will be recruited for the intervention phase.

### Recruitment {15}

Persons found to be hypertensive during baseline survey will be noted and contact address taken including the phone contact. Subsequently, they will be invited to the primary health facility in their neighbourhood for recruitment into the study (Intervention arm). In addition, the trained CORPs will screen and refer community members found to have high blood pressure to the primary healthcare facilities in the community (intervention arm) or health facility of their choice (control arm). The CORPs will carry out sensitization campaign in the community through visits to community stakeholders including the community chiefs, heads of religious bodies, leaders of artisans, head of community structures such as Chairman of Landlord Associations, and talks in churches, mosques and community meetings to intimate them of the programme and conduct blood pressure screening. The CORPs shall be provided with identification poster for display in front of their houses to indicate that blood pressure screening service is available. If the required number of hypertensive patients to be recruited in a community is not achieved, the neighbouring community may be co-opted and the CORP mobilized to cover the community as well. At the health facility, patients presenting with other ailments or walk-in patients or self-referred patients who present at the health facilities found to be hypertensive shall be requested to participate in the study. The physician/healthcare worker will confirm diagnosis and commence antihypertensive medications.

## Assignment of interventions: allocation

### Sequence generation {16a}

The unit of randomization will be the community. A total of 10 communities (five from each LGA) will be randomly selected from the list of communities from a ward obtained from the Local Government secretariat. The selected communities from each LGA will be grouped into two non-contiguous groups based on geographical distance between communities. One group will be randomly assigned to intervention and another to control. This will ensure that intervention communities are closed together but at far distance from control communities to minimize contamination. The final randomization of intervention and control communities will be done by a statistician who is not involved in the implementation of field activities. Assignment of communities to either the intervention or control arm will be done after the baseline survey. Given the nature of the intervention, it is not possible to blind the CORPs who will be involved in the delivery of the intervention.

### Concealment mechanism {16b}

Clusters (communities) will be randomly allocated to intervention and control group by the study statistician using a digital sealed envelope. This will be done after the collection of baseline data and just before the commencement of the intervention phase.

### Implementation {16c}

This is a cluster-randomized trial. Clusters or communities will be randomized and not individual participants. Allocation will be done by the statistician while participants will be enrolled by CORPS and health workers at the primary health centres.

## Assignment of interventions: Blinding

### Who will be blinded {17a}

Given the nature of the intervention, it is not possible to blind the CORPs who will be involved in the delivery of the intervention. However, the interviewers who will collect data for final assessment and the statistician may be blinded.

### Procedure for unblinding if needed {17b}

The design is open label with only outcome assessors and the statistician being blinded so unblinding will not be applicable.

## Data collection and management

### Plans for assessment and collection of outcomes {18a}

Standard operating procedures will be developed for each of the data collection activities to guide the research assistants and standardized the collection methods. Survey interviews will be conducted by 10 trained interviewers who will be graduates of tertiary institutions with experience in conducting community-based surveys and qualitative studies. Interviews will be conducted in secluded areas in the participants’ houses and at a convenient time of the day. Participants will be given a sachet of detergent to compensate for the time of the interview.

#### Quantitative data collection

Surveys will be conducted using the Research Electronic Data Capture (REDCap) software on Android mobile devices. The data collection tools will be developed using information from literature, consideration of variables in the study-specific objectives and the conceptual framework, researchers’ knowledge of the topic and adaptation of questions from past related studies. A semi-structured question will be drafted and face validated by experts to ensure the questions are adequate to provide answers to the study objectives. The draft questionnaire will be pre-tested in a community not selected in each of the six LGAs to ensure that questions are clear, comprehensible by participants, concise and consistent in a local government different from the ones selected into the study. The Cronbach alpha acceptable value will be set at 0.7 and above. The questionnaire will be translated into the local language and back translated into English to ascertain the correctness of the translation.

The survey questionnaire will be divided into the following sections: (a) demographic characteristics, (b) health status of the participants including, presence of chronic disease(s), knowledge of their blood pressure status, drug history, (c) health-seeking behaviour, (d) disease screening practices, (e) lifestyle including dietary habit, (f) knowledge of hypertension, risk factors and its control including sources of information, (g) cost of treatment of hypertension among those with hypertension, (h) willingness to accept CORPs to screen for hypertension and carry out follow-up care for hypertension in the community

#### Clinical and anthropometry measurements

##### Blood pressure

Blood pressure will be measured using a fully automated digital device, the OMRON digital sphygmomanometer (OMRON HEM-712C). Interviewers will be trained to use the device according to the manufacturer’s recommended protocol, and BP will be measured using recommended methods and categories from standard guidelines such as the World Health Organization-International Society of Hypertension Guidelines for the Management of Hypertension. The first blood pressure reading will be measured on the left arm with the participant in a seated position after resting for at least 5 min. The second and third readings will be taken about 2-min interval. In this study, hypertension was defined as average of two measurements of systolic and/or diastolic BP (2nd and 3rd readings) that is ≥140/90 mmHg in any adult according to the Joint National Committee JNC7 classification or self-reported previous diagnosis of hypertension and treatment of hypertension with antihypertensive medication taken in the past 2 weeks [34]. Participants with elevated blood pressure will be referred to a health facility for further assessment and confirmation of the BP status.

##### Electrocardiogram examination

The electrocardiogram (ECG) is listed among the routine investigation for hypertensive patients according to international guidelines. It has been used for the evaluation of subclinical cardiac damage in uncomplicated hypertensive patients [35]. The outcome could help with stratification of cardiovascular risk in hypertension. In this study, individuals confirmed to have high blood pressure shall have electrocardiogram (ECG) examination at baseline and endline. Those with abnormality shall be referred to a cardiologist in the nearest hospital.

##### Behavioural risk factors

The WHO STEPwise data collection tool for surveillance of non-communicable diseases will be adapted to gather information on behavioural CVD risk factors including smoking, alcohol drinking and dietary habits [36]. Questions will probe on current and past smoking as well as current and past alcohol drinking. Dietary assessment will include intakes of fruits, vegetables, salt, use of vegetable cooking oil and consumption of whole grains.

##### Anthropometric measurements

Anthropometric measurements will include weight, height and waist circumference taken using standard procedures. Briefly, all anthropometric measurements will be taken with the participant wearing light clothing and without shoes within the participant’s household compound. Body weight will be taken to the nearest 0.1 kg using a validated SECA digital scale placed on flat ground. Height will be taken in a standing position with heels perpendicular to the portable stadiometer, measured to the nearest 0.1 cm. BMI will be calculated as body weight divided by height squared (kg/m^2^). Overweight will be defined as BMI ≥ 25 kg/m^2^ but < 30 kg/m^2^ and obesity is defined as BMI ≥ 30 kg/m^2^ [36]. Waist circumference will be measured and recorded to the nearest 0.1 cm using a non-stretchable measuring tape at the mid-point between the lower margin of the last rib in the mid-axillary line and the iliac crest according the WHO guidelines [37]. Abdominal obesity will be defined as waist circumference ≥ 102 cm. Waist-to-hip ratio will be calculated and a value of less than 0.85 is considered as normal for women and less than 1.0 for men is normal.

##### Wealth index

The household wealth index of the participants will be generated as described in the demographic health survey using data on ownership of household items such as refrigerator, bicycle, radio, television, sofa, telephone, car and house ownership, construction materials (floor, walls and roofing materials); source of water supply for home use and drinking; source of fuel for cooking and lighting; and having working electricity and sanitation facility [38].

## Qualitative data collection

The guide will be developed for the focus group discussion (FGD) and key informant interview (KII). A pre-tested guide will be used to facilitate the FGD sessions and KII interviews. The sessions will be audiotape recorded after obtaining consent from the participants. The FGD will be conducted by a team of 2–3 research assistants—facilitator, note-taker / recorder. Snacks will be provided during the sessions.

Key informant interviews will be conducted among key people in the community, head of facilities, the physician, or health workers involved in the management of hypertensive patients. The focus group will comprise 6–12 homogenous community members. Two research assistants will facilitate the session. Participants will be provided incentive to compensate for the time at the FGD.

## Development of intervention materials

Results from the preliminary analysis of the baseline study data will help to identify gaps in the awareness, knowledge, perception, attitude and preventive practices regarding hypertension. This information will be used for the development of the intervention materials by the team members. These materials include (i) training manual for CORPs and health workers, (ii) information education communication/behaviour change communication (IEC/BCC) materials including healthy lifestyle hand bills, hypertension treatment guide, (iii) home-based follow-up care guide, (iv) standard operating procedures, (vi) short messaging system (SMS) messages.

## Training manual and guides

Training manuals will be designed adopting the relevant sections in the WHO training manual for the prevention of NCDS and health lifestyle [39] and manuals shared by researchers who have conducted similar studies. The training topics will include definition of hypertension, risk factors, detection and management, blood pressure screening procedures and interpretation of readings, anthropometric measurements, ethical issues, community mobilization and effective communication for behaviour change, health education on hypertension and healthy lifestyle and follow-up care for hypertension among others. Graphics and pictorials will be used as appropriate. The manual will be face validated by two researchers involved in hypertension studies. In addition, guides will be developed using algorithms and adapting the National Hypertension Control guidelines.

## Health education materials

The health education materials will be developed by team members using illustrations from literature, adapted from IEC materials shared by other researchers and drawn by an artist. The draft will be presented to CORPs and health workers for their input after which it will be face validated by selected community members before final production. The materials will be in form flyers/brochures with messages on hypertension as silent killer, the risk factors and how to prevent them such as reducing salt intake, reducing or maintaining normal/ideal body weight, smoking cessation, reducing alcohol intake, increasing consumption of fruits and green leafy vegetables, using vegetable cooking oil and engaging in exercise. Training modes will include didactic lecture, case study, role plays and practical sessions in the field.

### SMS message

The content of reminders will be prepared based on literature search and consultations with communication expert. A SMS reminder content of about a minute duration will be designed. The message will be prepared in English and translated to local languages (Hausa, Igbo and Yoruba) version; thereafter, back translated to English to verify accuracy of the translation by an expert in both English and the local languages. It will be validated and pre-tested among 10 selected members of communities not included in this study.

### Plans to promote participant retention and complete follow-up {18b}

Participants will be provided adequate information on the project and their role as well as what is expected of them emphasizing completeness of follow-up. It will be ascertained at time of recruitment that the participant will be resident in the community for at least a year. The participants will be linked to the CORPs in their community who will provide follow-up care and visit them to check on adherence to clinic follow-up visit. The participant is recruited from the catchment area of the clinic; hence, it is assumed that this will encourage clinic as distance to clinic will not be a barrier to follow-up visit. The proportion of participants who visit the clinic and those who had home follow-up visit by CORPs as scheduled shall be measured at the end of the 6-month follow-up visit.

### Data management {19}

The research assistants (data collectors) will upload the data collected on their android phone daily into the REDCap database server from where the data manager will retrieve and review the data daily for quality assurance and control. The data manager will provide feedback on entered data early the following morning they were collected so that research assistants can correct wrong entries and fill gaps or missing data as necessary.

For the qualitative data, the audio recording will be carried out using a good quality tape recorder and research assistants will ensure it is functioning, label the sides and pre-record to test before use for the sessions. The tapes will be kept in locked boxes to prevent destruction and for confidentiality. Analysis will be carried by two investigators independently. Where they differ, the transcribed notes will be revisited and analysed again till there is a consensus.

### Confidentiality {27}

#### Handling and storage of data

The confidentiality of all participants information will be protected in accordance with national and international data protection laws. The study team will assure that participants’ anonymity will be maintained and that their identities are protected from unauthorized parties.

Participants will be assigned unique code as identification numbers for data collection. All information will be collected using the unique study participants’ code numbers except for consent forms, which will bear the name and signature of the participant. Only the statistician and data manager have access to the files in the REDCap. Documents (i.e. participant signed ICF, medical records, case record forms) will be maintained in strict confidence in passworded files on the computers and locked cabinets in the office. Any reports or publications or scientific presentations will not contain any participants’ identifying information. The data will be in storage for at least 5 years and any request to use them for secondary data analysis shall be made to the principal investigator after agreeing to conditions for data sharing and all data will be de-identified.

### Plans for collection, laboratory evaluation and storage of biological specimens for genetic or molecular analysis in this trial/future use {33}

Ready-to-eat food samples will be collected randomly at unannounced visit to a participant’s home twice (including one weekend day) during the study and kept frozen in a sterile bottle. Biochemical analysis of food sodium content will be carried in certified laboratories such as the International Institute of Tropical Agriculture, (IITA) Ibadan, Nigeria.

Blood samples for estimation of urea, creatinine and lipid profile will be collected according to standard laboratory guideline under sterile conditions at recruitment and at the end of the 6-month intervention period in the health facility. Urine samples will also be collected and analysed using Uristix® at the same time. Blood sample analysis will be at the Chemical pathology laboratory of a tertiary institution in the study states.

## Statistical methods

### Statistical methods for primary and secondary outcomes {20a}

Data analysis will be carried out using Stata MP version 14 based on the CONSORT 2010 guideline [40]. Intention-to-treat analysis will be applied. Continuous variable will be presented as mean and standard deviations (SDs) or medians with their 25th and 75th percentiles when the distribution of the data does not follow a Gaussian distribution. Categorical variables shall be presented as frequencies and percentages.

Baseline characteristics of participants in the intervention and control arms will be compared to assess balance and identify any variable that need to be adjusted in the final analysis. The primary outcome is achievement of blood pressure control at 6 months post-intervention. The effect of intervention within each of the study groups will be carried out comparing baseline and 6 months post-intervention using McNemar test. Depending on the distribution of baseline characteristics, random effect logit model will be fitted to estimate treatment effect size comparing the two groups. Similar analysis will be done for categorical secondary outcomes such as treatment adherence.

Cost-effectiveness analysis will also be carried out using the ingredient approach [41].

#### Qualitative data

The audiotape recordings from the FGD and KIIs will be transcribed verbatim and translated into English. This will be analysed by content analysis using NVIVO. The text will be entered into NVIVO and codes and themes on acceptability and performance of the CORPs and quality of the intervention will be generated.

Qualitative data analysis of qualitative data from the FGDs will be done using Nvivo 9® software and manual coding. The audio recordings from FGDs will be transcribed verbatim and translated into English. Caution will be taken to ensure that no data will be lost during translation. The text data will be entered into Nvivo 9® software and analysed for themes on perceptions about quality and acceptability of the CHW intervention in the community

### Interim analyses {21b}

Interim analysis will not be carried out.

### Methods for additional analyses (e.g. subgroup analyses) {20b}

Numerical secondary outcomes such as mean change in systolic blood pressure will be analysed using two approaches. First, a difference-in-difference method [42] would be employed to compare mean SBP between baseline and 6-month post-intervention. This will be an assessment of within-group changes in blood pressure. Secondly, a linear mixed model will be implemented to determine the treatment effect as a measure of between-group comparison of intervention and control arms.

### Methods in analysis to handle protocol non-adherence and any statistical methods to handle missing data {20c}

The use of CORPS who are based in the study communities and monthly home visit will help to minimize the occurrence of missing data and losses to follow-up. During analysis, sensitivity analyses will be carried out to assess the patterns of missing data and loss-to-follow-up so that appropriate decisions can be taken on whether multiple imputation methods need to be employed. The level of significance will be set at 5%.

### Plans to give access to the full protocol, participant-level data and statistical code {31c}

The team plans to grant public access to the study protocol and de-identified data set after due request and commitment to adhere strictly to ethically consideration. This will only be available after 3 years of study completion.

## Oversight and monitoring

### Composition of the coordinating centre and trial steering committee {5d}

The team will constitute its own monitoring team to monitor the implementation of the project. Monitoring checklist will be developed for this exercise. Monitoring visits shall be carried out every 2 months or more frequently if indicated. The team shall hold review meetings with field supervisors weekly to check process, address challenges and motivate. The field supervisors shall submit weekly reports on activities of the field and shall be required to call if there is any emergency or disruptions in the field. The investigators shall hold team meetings monthly or more frequently, if necessary, to track the implementation process. A monitoring team shall be constituted from among the investigators to assess trial progress and safety of participants.

This study coordinating centre is the Epidemiology and Biostatistics Research Unit (EBRU), Institute for Advanced Medical Research and Training, College of Medicine, University of Ibadan. The institute comprises several research laboratories. Members of EBRU are diverse in expertise and are involved in various research projects including clinical/field trials and operational research. The investigators on the team to carry out this proposed study comprise of seasoned epidemiologists, seasoned primary healthcare specialist, nutrition expert, statistician, social scientist, cardiologist, policy experts, and a health economist.

The unit has been in existence for upward of 10 years and are well experienced in clinical/field trials and operational /implementation research. These include studies on home management of malaria, intermittent preventive treatment (IPT) use in pregnancy, artemisinin combination therapy (ACT) effectiveness and feasibility studies, intervention to improve ITN use among under-fives and pregnant women, neglected tropical diseases—helminthiasis, hypertension in adults and children, salt intake and hypertension among school children and other community-based studies. The team has carried out many of their research works in collaboration with the Federal Ministry of Health and State Ministries of Health.

The College of Medicine has an IT unit that supports researchers on issues related to technology and M-health. It has the REDCAP software that will support this project in kind with data capturing. The unit shall provide advice and purchase the Cloud system, the SMS messaging App to be used for the IT component of this study from the grant money to be provided by TETFund.

## Roles and responsibilities of team members

The study shall be carried out by a multidisciplinary team comprising experience researchers, experts in relevant disciplines, young faculty, and mentees. They will be involved in the planning of the different segments of the study implementation and provide day to day support for the trial. The team plans to meet monthly and occasionally have ad hoc meetings to deliberate on burning issues or unanticipated challenge on the field. In addition to the Statutory meetings, the team will have seminars to present current issues in the study topic areas, workshops for development of data collection tools, data analysis and interpretation, dissemination and report writing among others. These will occur prior to the activities to plan and as the activities are completed to appraise and plan for future activities. Details of the roles of the team members are as below.

1. Principal Investigator

An epidemiologist, Public Health and Family Physician. She will oversee the entire project and research activities. She will monitor, supervise and direct the research and coordinate the activities in the three states (study sites) and two arms of the study, including data management. She will organize team meetings, disseminate updates of project periodically and play host to other key persons at meetings. She will visit the study sites for oversight function and attend advocacy meetings at local, state and national levels on behalf of the study team.

2. Two cardiologists who will oversee the clinical aspect of the study and ensure the health facilities are adequately equipped to management hypertension. They will oversee the training of the health workers. Each of them will coordinate and supervise research activities in Abia and Kano States Sites respectively.

3. A Health Education Specialist and Nutrition expert who will oversee the health education and counselling on nutrition and healthy lifestyle, will co-train the CORPs, health workers and the research assistants. He will oversee advocacy visits, development of the BCC/IEC materials, community mobilization and entry.
4. A Medical Statistician. He will support the team with data management and train the field staff on data collection and data cleaning. Supervise the data manager and be custodian of the data set on REDCap. He will advise on questionnaire development, selection of study communities, randomization of study arms and data analysis statistical procedures and contribute to manuscript writing.
5. Two Epidemiologists and Community Physician who will support the team with advocacy, participate in selection and training of research assistants and CORPs and provide support to the field supervisors, community mobilization and entry. They shall contribute to data collection tool development and support the statistician and data manager in data analysis and interpretation.
6. A health policy specialist who will support the team regarding advocacy and advice on policy matters. He shall support training and supervision of the field workers. He will support the development of the questionnaire especially in the area of costing and health system utilization as well as health facility capacity and capability. He shall play a vital role in the dissemination process and writing of policy brief.

### Composition of the data monitoring committee, its role and reporting structure {21a}

A data monitoring committee was not considered as this was a low-risk intervention.

### Adverse event reporting and harms {22}

The interventions are mostly behavioural. Therefore, no serious adverse event is anticipated. However, any adverse event due to use of antihypertensive medications will be addressed as routinely done in clinics.

### Frequency and plans for auditing trial conduct {23}

The study steering committee will meet every month to review the trial progress and address any implementation challenges as they arise.

### Plans for communicating important protocol amendments to relevant parties (e.g. trial participants, ethical committees) {25}

The ethical review committee has a standing guideline as to how to apply for amendment in a protocol. This will be followed if the need should arise. The grant supporting body, Tertiary Education Trust Fund (TETFund), National Research Fund (NRF), shall be notified through a letter and the approved amended protocol after the ethical committee has approved the amendment. The study participants will be informed duly as the amendment affects their participation. This may be done at individual or group level. Trial registries shall be notified using the registry’s guideline.

### Dissemination plans {31a}

We would make our results available to the community of scientists with interest in this field in order to prevent unintentional duplication of the project. We would welcome collaboration with other implementing partners who could make use of information obtained from our project for formulation of policies. We shall disseminate by doing the following:

## Dissemination to the local authorities

The findings of this cluster-randomized controlled trial shall be presented and communicated in the appropriate local governments in each state to key stakeholders in the community, local government as well as the States Ministry of Health and the Federal Ministry of Health. In addition, the group shall on an annual basis organize a 1-day hypertension awareness programme during which risk factors for hypertension and preventive measures shall be discussed.

## Dissemination to policy makers

So also, our findings will be presented relevant key stakeholders in the States Ministry of Health and the Federal Ministry of Health. This will be useful in formulating appropriate policy in line with the scale up of response to non-communicable diseases (NCD) in Nigeria. They will be encouraged to engage with relevant bodies and implementing partners providing CVD-related programmes.

## Dissemination to the scientific community

We will present and discuss our findings at the institutional, national, continental and international scientific meetings. The group will publish newsletter annually. The newsletter’s intent is to disseminate new information from our research. The activities and discoveries of the project will be allocated 40% of the newsletter’s coverage. In addition, the group will also create a website where information about project as well as the diseases of interest shall be posted regularly. The language shall be made simple and short and the primary audience is the general public.

## Discussion

This study is one of the few ongoing studies targeting hypertension control in Nigeria with the intention of involving community in the management through ownership of intervention process that will foster sustainability. It is a multi-centre study including three states representing the three major ethnicities in Nigeria, the Hausa, Igbo and Yoruba. The study is based on community-clinical linkage using task sharing concept of healthcare delivery in underserved urban and rural communities. Nigeria, a low-resource setting, is characterized by shortage of physicians and health workers in urban and especially rural areas [43]. Task sharing and task shifting is defined as the allotment of tasks usually performed by highly trained healthcare workers (doctors and nurses) to less specialized health workers, who often have less education and training [44, 45]. It has been demonstrated that patients can effectively be assessed for CVD risk and managed in primary care facilities by non-physician health workers in low-resource settings [46]. An adequately trained local workforce with the capacity to initiate and sustain CVD prevention and management programmes would be a good complement to formal healthcare delivery system. In this study, we seek to develop a team of local healthcare human resource to provide sustainable CVD prevention and hypertension follow-up care in the community. The intervention package to be deployed will focus on improving knowledge of hypertension, early diagnosis of hypertension and identification of those with risk factors of cardiovascular diseases, promoting healthy lifestyle for reduction of CVD risk factors in the community.

The study will be one of the first few studies in Nigeria that will be evaluating the effectiveness of training community-oriented resource persons to screen for hypertension and provide health education services on the clinical outcomes of hypertensive patients comparing the intervention and control arm. This will be achieved by health education and awareness campaign, task sharing using the community-oriented resource persons (CORPs) in the community for home-based follow-up care and building health facility human and infrastructure capacity.

The high prevalence of hypertension and the increasing trend of early onset is a major concern in the development of a nation as the young and active workforce population are being incapacitated and worst still depleted. The Nigerian government in a bid to combat poverty, hunger, disease, illiteracy, environmental degradation and discrimination against women adopted the United Nations sustainable development goals (SDGs), which are interdependent and influence health and vice versa [47]. However, efforts in the various sectors including poverty reduction (SDG 1) have not yielded the desired results or outcome and have stalled the achievement of other SDGs. This proposed study addresses achieving good health and well-being, which is goal 3 of the SDGs. It stands to provide evidence-based information for the adoption of an effective strategy in the control of hypertension with the goal of reducing mortality and morbidity as well as improving the workforce for nation development. The study will also empower the communities to take ownership of the intervention for sustainability of the project.

The study is not without limitation. This study is coming up in a pandemic era and could be disrupted at some points in the implementation. These include possibility of delay in commencement, health facility attention to the project may be distracted and patients’ adherence may be hindered. The team shall endeavour adherence to all the COVID-19 preventive measures. The CORPs and health facilities will be provided with face masks and hand wash and sanitizers. Field activities will be suspended should there be a lockdown till when eased.

Basing the status of blood pressure on readings on one single visit during survey may not show the true prevalence of hypertension in the communities with likelihood of overestimation. However, using an average of three BP measurements with resting periods in between is expected to minimize this.

The population characteristics of the urban and rural areas could differ especially with age and sex bearing in mind population migration factors and dynamics. This may distort the true effect of the intervention on outcome as CVD risk factors varies in these groups of people. This will be adjusted at analysis using multilevel analysis with random effect model. Bias in the intervention study will be minimized by randomization. The possible contamination between control and intervention arm will be reduced by grouping/clustering the communities before randomization and ensuring buffer zones exist between them to limit diffusion.

Cluster-randomized design is appropriate for this study which by nature is evaluating the effectiveness of an interventions in a “real-world setting” [48], and meant to provide health education at both community and individual levels. In addition is the fact that it is also an effective way to avoid contamination. The limitations associated with its use such as prevention of interference shall be giving much attention at analysis.

In conclusion, this study stands to provide evidence-based information for scaling up task sharing as well as community-clinical linkage in the control of hypertension in Nigeria. It is expected that this intervention will bring about early detection of hypertension, reduction in mean blood pressure and an increase in the proportion of patients with controlled blood pressure. In addition, we expect an improvement in the mean quality of life score; healthy lifestyle and competent CORPs as well as the reduced mean cost of treatment of hypertension in the intervention arm of the study. All of these will inform policy review to guide the control of hypertension and reduction in cardiovascular diseases risk in Nigeria.

## Trial status

The study protocol version number is 3 (December 01 December 2019). The study commenced in August 2020 and expected to be completed in July 2022. The recruitment start date for the cluster RCT is September 2021 and end date is February 2022. Formative phase has been concluded. The recruitment for the intervention has not started.

## Abbreviations

BMI: Body mass index
CORPs: Community-oriented resource persons
CVDs: Cardiovascular diseases
CVE: Cardiovascular event
DBP: Diastolic blood pressure
DM: Diabetes mellitus
EBRU: Epidemiology and Biostatistics Research Unit
FGDs: Focus group discussions
HDSS: Health and demographic surveillance system
HICs: High-income countries
LGA: Local Government Area
LMICs: Low-and middle-income countries
IAMRAT: Institute for Advanced Medical Research and Training
NCDs: IEC/BCC: Information education communication/behaviour change communication
iCCM: Integrated community-based case management of febrile illness
KII: Key informant interview
NCD: Non-communicable diseases
RedCap: Research Electronic Data Capture
SBP: Systolic blood pressure
SMS: Short message services
SSA: Sub-Saharan Africa
WHO: World Health Organization
